# Field Tested: Grasslands Won’t Help Buffer Climate Change as CO2 Levels Rise

**DOI:** 10.1371/journal.pbio.0030329

**Published:** 2005-08-09

Serious gardeners know a healthy harvest depends on the right mix of ingredients at the right time. Nitrogen, which spurs leafy growth at the expense of flowers, works best soon after plants emerge. Phosphate, which promotes blossoms, should come later. Indoor growers often gas their plants with CO2 to boost growth, development, and budding. The *really* serious indoor gardener can pay $599 for a microclimate controller that automatically regulates temperature, humidity, CO2, lighting, and irrigation. 

These components are just as important to natural ecosystems as they are to the well-tended garden. But their composition in the atmosphere and soil is changing at an unprecedented rate. Rising concentrations of CO2 and other greenhouse gases—which have increased apace with global agricultural and industrial development—are linked to warmer temperatures and changes in precipitation patterns. The by-products of industrial and agricultural operations have also increased nitrogen concentrations, saturating systems in highly polluted areas. 

Plants use CO2 (and water and light) during photosynthesis to make sugar and grow. A plant absorbs CO2 through its leaves and takes up nitrogen (often in the form of nitrate) and other soil nutrients through its roots. Increasing CO2 concentrations can facilitate plant growth by increasing the rate of photosynthesis, though this effect varies depending on the photosynthetic pathway the plant uses. Trees, for example, use a different pathway than most tropical grasses. Higher carbon inputs can also trigger more efficient nitrogen use. Because plants are primary producers, at the base of the food chain, major shifts in the global cycles that support them will have wide-ranging consequences.

To observe how a natural system might respond to these changes over the long term, a team led by researchers from the Carnegie Institution of Washington and Stanford University created an experimental system in their Northern California backyard. Jeffrey Dukes, Christopher Field, and their colleagues treated grassland plots to every possible combination of current or increased levels of four environmental factors—CO2, temperature, precipitation, and nitrogen influx—to simulate likely regional changes over the next 100 years. Previous studies have tested natural systems’ responses to one or two of these changes, but none has tested the long-term, simultaneous impacts of each.[Fig pbio-0030329-g001]

**Figure pbio-0030329-g001:**
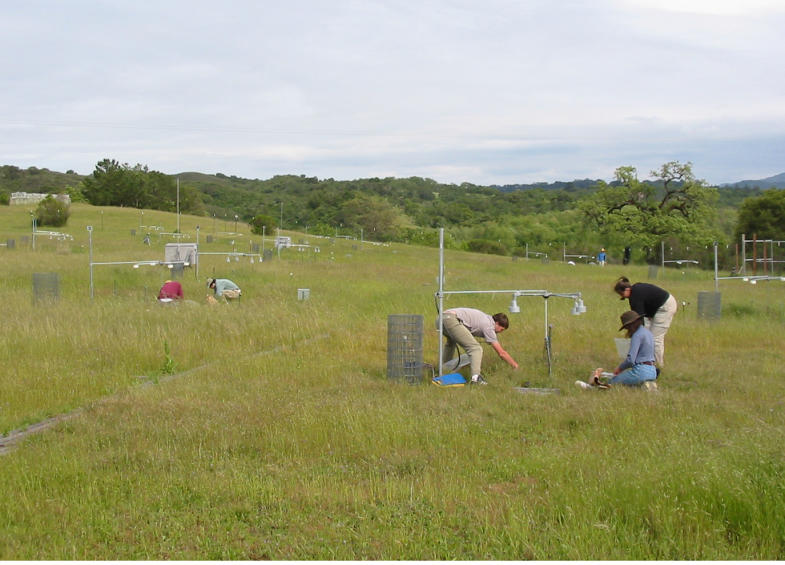
Researchers work on some of the 36 plots that lie below four infrared heaters in the Jasper Ridge Global Change Experiment. Total plant growth in grassland plots like these rarely responded to changes in climate or atmospheric CO_2_ concentration

The strongest effects on grassland production came from elevated levels of nitrogen (which typically reaches a fertilization limit). Elevated temperature, precipitation, and, surprisingly, CO2, had minimal impacts. These results suggest, the authors argue, that California grasslands, and ecosystems that respond similarly, are not likely to help buffer the rate of climate change by acting as a carbon “sink”—slowing the rise of CO2 levels by storing more carbon in new growth. It’s thought that ocean and terrestrial ecosystems have stored nearly half the carbon emissions produced by humans since the industrial revolution. If it turns out that other natural systems also fail to sequester as much carbon as scientists once thought, atmospheric CO2 concentrations will rise even faster than expected—with serious implications for future climate change. 

The experiments were part of the Jasper Ridge Global Change Experiment (JRGCE), which started on Stanford’s 1,200-acre biological preserve in 1997. Since 1998, this grassland ecosystem has been outfitted with an ecologist’s version of a microclimate controller (complete with CO2 pumps, heaters, and irrigation tubing) and subjected to experimentally controlled atmospheric, climatic, and nutrient conditions. (This study examines the experiment’s first five years.) To quantify the grassland response to these treatments, the authors estimated net primary production, or NPP (the amount of carbon left over after cellular respiration) by measuring shoot and root growth in 36 circular plots scattered across roughly two acres. Four control plots experienced the natural variations of California’s Mediterranean climate.

Overall, increased rainfall, warming, and elevated CO2 had little effect on NPP. (More rain triggered shoot growth but stunted root growth, so NPP wasn’t affected.) In some experimental treatments and years, elevated CO2 actually reduced grassland production. Increased nitrate, on the other hand, led to shoot and root growth imbalance, with shoots growing faster than roots. And this added nitrogen “strongly increased” NPP in every year but one. These results suggest that increasing concentrations of atmospheric CO2 are not likely to increase growth of the roots and leaves of plants in this grassland. Why not?

One possibility involves phosphorus. High levels of CO2 and nitrogen can reduce phosphorus concentrations or limit its uptake in these plants. Ongoing JRGCE experiments are exploring how this and other factors—such as grazing or shifts in seasonal events—might limit the growth effects of CO2. 

Because grasslands and forests operate in complex feedback loops with both the atmosphere and soil, understanding how ecosystems respond to global changes in climate and element cycling is critical to predicting the range of global environmental changes—and attendant ecosystem responses—likely to occur. Ecosystem responses may well vary according to their composition and location. By studying natural systems over multiple years on an ecosystem scale, experiments like JRGCE offer a valuable tool for simulating predicted global changes and assessing their likely impacts, region by region.

